# Lymph node metastasis in suprasternal space and intra-infrahyoid strap muscle space from papillary thyroid carcinoma

**DOI:** 10.1186/s40463-020-00461-2

**Published:** 2020-08-28

**Authors:** Qianqian Yuan, Jinxuan Hou, Yiqin Liao, Lewei Zheng, Fang Lu, Kun Wang, Gaosong Wu

**Affiliations:** 1 Department of Thyroid and Breast Surgery, Zhongnan Hospital of Wuhan University, 169 Donghu Road, Wuhan, Hubei People’s Republic of China 430071; 2 Department of Thyroid and Breast Surgery, Tongji Hospital of Tongji Medical College of Huazhong University of Science and Technology, 1095 Jiefang Avenue, Wuhan, Hubei People’s Republic of China 430030

**Keywords:** Thyroid carcinoma, Surgery, Central compartment neck dissection, Recurrence, Suprasternal space

## Abstract

**Background:**

This study was performed to evaluate the clinicopathologic characteristics of **L**ymph **N**ode metastasis between investing layer of **C**ervical fascia and deep fascia of infrahyoid strap **M**uscles (LNCM) in papillary thyroid carcinoma (PTC).

**Methods:**

Retrospective review of patients with PTC who underwent thyroidectomy and central compartment neck dissection (CND) from January 2016 to January 2018 was performed in two tertiary referral academic medical centers. A total of 2104 consecutive patients with PTC who underwent thyroidectomy and CND were included in the retrospective review. The LNCM was resected as a separate specimen by the surgeon and the clinicopathologic characteristics of the patients were recorded. Multivariate logistic regression analysis was performed to identify risk factors for LNCM metastasis.

**Results:**

Of 2104 PTC patients, 451 patients (21.4%) had lymph nodes in the LNCM. Among them, 68 (15.1%) cases were confirmed to be positive in the LNCM. In total, the metastasis rate of LNCM in PTC patients was 3.2% (68/2104). Univariate analysis revealed that the metastasis of LNCM were more likely to have a primary site in the inferior pole, extrathyroidal extension (ETE), central cervical metastasis, level III and level IV metastasis. Multivariate analysis further showed tumor location in the inferior pole, ETE, level III and level IV metastasis conferred a significantly increased odds ratio for LNCM metastasis.

**Conclusion:**

Attention should be paid to the lymph tissue in the LNCM for PTC patients, especially in presence of a primary site in the inferior pole, ETE, level III and level IV metastasis.

## Introduction

Patients with papillary thyroid carcinoma (PTC) have a favorable prognosis with central neck locoregional recurrence varying from 0 to 20% [1]. The goal of a prophylactic or therapeutic central compartment neck dissection (pCND or tCND) is to decrease the incidence of local recurrence by removing all lymphatic tissue within the levels VI and VII compartments, which are generally the first and the most commonly involved with metastasis [2]. For patients without evidence of lymph node metastasis on preoperative evaluation, the additive value of a pCND at the time of thyroidectomy is controversial. Some authors advocate pCND, considering high rate (24–88%) of occult metastatic nodal disease in cN0 PTC [1], while other authors consider that there is no high-level evidence in favor of pCND [3]. The performance of pCND is dependent on the weight given to the risks and benefits of pCND [4]. Considering the oncologic benefits of CND and the risks of a repeat neck operation, performing pCND is recommended to every patient in China [5, 6].

Although American Thyroid Association (ATA) guideline has defined the boundary of central neck compartment, there is also significant variability in terms of the extent of CND. In routine clinical practice, CND can range from sampling a few nodes in the paratracheal region to a complete clearance from left carotid artery to right carotid artery and down to and including the upper mediastinum [7]. Owing to the variant extent of CND, some central compartments are easily to be neglected. For thyroid carcinoma patients with specific clinicopathologic characteristics, incomplete lymph node dissection may result in increased recurrence, reoperation, and reoperation-associated complications [8]. **L**ymph **N**ode between investing layer of **C**ervical fascia and deep fascia of infrahyoid strap **M**uscles (LNCM) has not been reported. The LNCM compartment is defined as follows: superiorly by the hyoid bone, laterally by the carotid arteries, anteriorly by the investing layer of cervical fascia, and posteriorly by the deep fascia of infrahyoid strap muscles. LNCM space includes suprasternal space and intra-infrahyoid strap muscle space.

**Fig. 1:**
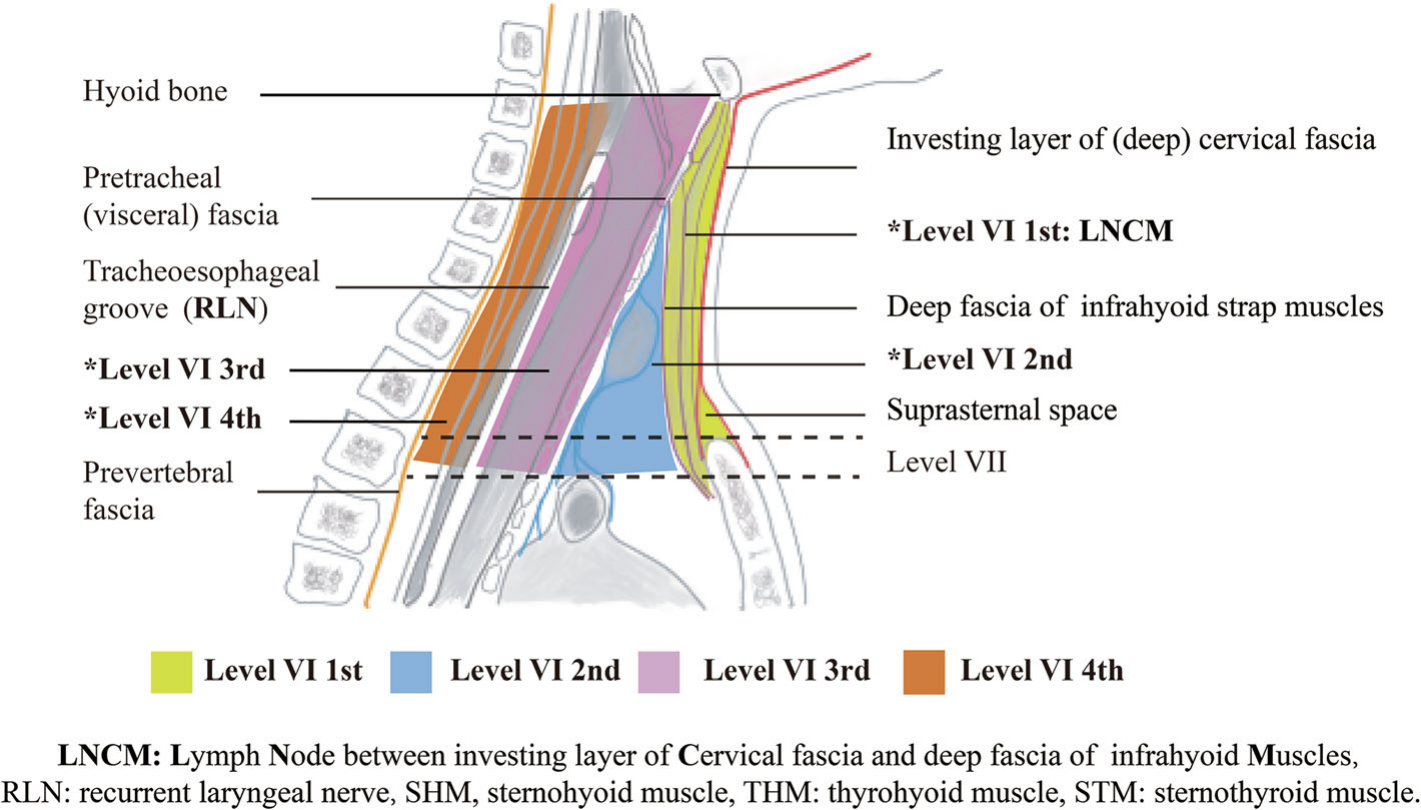
Four subdivisions (Level VI 1st, Level VI 2nd, Level VI 3rd, and Level VI 4th) of central neck compartment are divided by deep fascia of infrahyoid muscles, pretracheal (visceral) fascial, and right recurrent laryngeal nerve

Anatomically, LNCM is located anterior to the strap muscles. We consider that what is special about the concept of the LNCM is that it is belong to level VI but is an easily overlooked anatomical area by a strap musculature involving the sternohyoid and sternothyroid muscles during selective or modified neck dissection. Although the metastasis in LNCM was seldom, it did occur in some PTC patients with regional recurrence. As part of LCNM, suprasternal space metastasis for thyroid cancer were investigated in three studies [9–11]. Thus, we routinely detected the suprasternal space and intra-infrahyoid strap muscle space (Fig. 1). This study was performed to identify the clinicopathologic characteristics and indication for lymph node metastasis dissection in the LNCM.

## Materials and methods

### Patients

A retrospective review from the clinical and histopathology database of two tertiary referral academic medical centers, Tongji Hospital of Huazhong University of Science and Technology and Zhongnan Hospital of Wuhan University from January 2016 to January 2018 were conducted. In the institutions, preoperative examinations consisted of a thorough physical examination, neck ultrasound, a clinical evaluation of thyroid nodules and neck lymph nodes. Fine needle aspiration cytology (FNAC) were performed in patients who were suspected thyroid nodules or lymph node. With a pathological confirmation of PTC, all the patients received a thyroidectomy with CND. Accordingly, a pCND was performed for cN0 patients, and a therapeutic CND was performed for cN1 patients. The inclusion criteria for the patients were as follows: (1) the clinical history completely recorded; (2) the LNCM was resected as a separate specimen by the surgeon; (3) PTC patients who underwent thyroidectomy plus CND with or without lateral neck dissection. A total of 2104 consecutive PTC patients were enrolled. The medical ethic committee of Zhongnan Hospital of Wuhan University approved the procedure and informed written consent was obtained from all patients.

### Surgical approach

All the operations were performed by the same senior surgeon (Gaosong Wu), with the patients under general anesthesia. Thyroidectomy was performed with a standard technique of fine capsular en bloc dissection and resection, from inferior pole to superior pole [12–16]. Intraoperative neuromonitoring was employed for all of the thyroidectomies [17]. Superior parathyroid glands were identified and preserved in situ, inferior parathyroid glands were protected in situ or autotransplanted in the sternocleidomastoid muscle according to three certain types based on their blood supply and location [14, 18].

After the incision of the investing layer of cervical fascia, the interval between sternohyoid and sternothyroid muscles and the space anterior to the sternohyoid muscle above the clavicle and the sternum were detected. If there was fibrofatty tissue Instead of the en bloc removal of the entire central lymph nodes, the LNCM was resected as a separate specimen if occurred. The presence or absence of lymph node metastasis was defined according to postoperative pathological reports. While dissecting paratracheal lymph nodes, intraoperative neuromonitoring was employed to detect RLN from distally to proximally, minimizing morbidity from injury to RLN during compartment nodal dissection. LNCM and other compartment lymphatic tissue were processed for routine hematoxylin and eosine (H&E) separately. The pathologic results were independently determined by two qualified pathologists, without any prior knowledge of the patients’ clinical data.

### Data collection and statistics analysis

To determine the relation between LNCM metastasis and clinicopathologic factors, such as age, sex, primary tumor site, lateral cervical lymph node metastasis, level VI metastasis, the chi-square test and Fisher’s exact test were used as appropriate. Multivariate logistic regression analysis was performed to identify risk factors for LNCM metastasis of PTC. *P* < 0.05 was considered statistically significant. All calculations were performed using SPSS 20.0 for windows. Post-thyroidectomy hypocalcemia lasting for more than 6 months was considered as permanent VCP. All patients were followed up every 3–6 months postoperatively.

## Results

### Patients detected with LNCM

After reviewing 2104 patients who underwent thyroidectomy plus CND with or without lateral neck dissection from January 2016 to January 2018, 451 patients (21.4%) were detected with LNCM and 1653 patients were absent of LNCM. The average tumor size of LNCM was 2.35 cm and the mean number of lymph nodes sampled from LNCM was 3.5, ranging from 0 to 9. Table 1 shows the comparison of clinicopathologic characteristics between the present LNCM group and the absent group. In univariate analysis, Hashimoto’s disease (*p* = 0.001), multifocality leisions (*p* < 0.001), the tumor located in inferior portion (*p* < 0.001), extrathyroidal extension (ETE) (*p* < 0.001), central cervical metastasis (*p* = 0.017), level III and level IV metastasis (*p* < 0.001) were significantly associated with high prevalence of LNCM.

**Table 1:** Univariate analysis of demographic and clinicopathologic factors for patients who had lymph nodes in LNCM compared to those who did not

Variables	Present (*n* = 451)	Absent (*n* = 1653)	*P* value
Age (mean ± SD)			
≤ 55/> 55	274/177	946/707	0.179
Gender			
Female / Male	247/204	886/767	0.659
Tumor size			
< 1.0 cm / ≥1.0 cm	121/330	448/1205	0.908
Coexistent thyroid			
Nodular goiter			
Yes / No	47/404	183/1470	0.695
Toxic goiter			
Yes / No	74/377	238/1415	0.287
Hashimoto’s disease			
Yes / No	208/243	614/1039	0.001
Tumor focality			
Multifocality / Unifocality	303/148	605/1048	< 0.001
Tumor location			< 0.001
Inferior portion	245	639	
Upper-Middle portion	206	1014	
Extrathyroidal extension			
Yes / No	241/210	725/928	< 0.001
Central cervical metastasis			
Yes / No	304/147	1013/640	0.017
Lateral cervical metastasis			
Level II			
Yes / No	80/371	257/1396	0.216
Level III			
Yes / No	229/222	542/1111	< 0.001
Level IV			
Yes / No	233/218	571/1082	< 0.001

*LNCM* Lymph Node between superficial layer of deep Cervical fascia and deep fascia of infrahyoid Muscles

### Patients with metastatic LNCM

Among a total of 451 patients with LNCM, metastatic LNCM was found in 68 (15.1%) patients. Table 2 compares the clinicopathologic characteristics between the metastatic LNCM group and the nonmetastatic LNCM group. Three hundred eighty-three patients were confirmed free of LNCM metastasis, 249 (65.0%) of them with clinically negative node performed pCND and 134 of them with clinically positive performed tCND. All the patients in the metastatic LNCM group performed tCND. Lateral neck dissection was performed in 31 (81.6%) cases in the metastatic LNCM group and 185 (48.3%) cases in the nonmetastatic group, all lateral neck dissection was therapeutically performed. Univariate analysis was performed for the 68 patients with and 383 patients without metastatic LNCM. Age at diagnosis, gender and tumor size, coexistent thyroid disease, tumor focality, and level II metastasis were not correlated with LNCM metastasis. Univariate analysis identified tumor located in the inferior pole, central cervical metastasis, ETE, level III and level IV metastasis as significant predictors of LNCM metastasis in our study population. Multivariate analysis further showed that tumor location, ETE, level III and level IV metastasis conferred a significantly increased odds ratio for LNCM metastasis (Table 3).

**Table 2:** Univariate analysis of demographic and clinicopathologic factors for positive LNCM

Variables	Metastasis (*n* = 68)	Non-metastasis (*n* = 383)	*P* value
Age (mean ± SD)			
≤ 55/> 55	40/28	234/149	0.724
Gender			
Female / Male	36/32	211/172	0.743
Tumor size			
< 1.0 cm / ≥1.0 cm	22/46	99/284	0.269
Coexistent thyroid			
Nodular goiter			
Yes / No	9/59	38/345	0.410
Toxic goiter			
Yes / No	7/61	67/316	0.140
Hashimoto’s disease			
Yes / No	172/184	36/59	0.070
Tumor focality			
Multifocality / Unifocality	53/15	250/133	0.040
Tumor location			< 0.001
Inferior portion	60	185	
Upper-Middle portion	8	198	
Extrathyroidal extension			
Yes / No	63/5	178/205	< 0.001
Central cervical metastasis			
Yes / No	54/14	250/133	0.022
Lateral cervical metastasis			
Level II			
Yes / No	15/53	65/318	0.311
Level III			
Yes / No	43/25	186/197	0.026
Level IV			
Yes / No	58/10	175/208	< 0.001

*LNCM* Lymph Node between superficial layer of deep Cervical fascia and deep fascia of infrahyoid Muscles

**Table 3:** Multivariate analysis of predictors for LNCM positivity

Variables	OR (95% CI)	*p* value
Tumor location	1.198 (1.056–1.703)	0.002
Central cervical metastasis	1.677 (0.149–3.078)	0.614
Extrathyroidal extension	1.108 (1.013–1.939)	0.042
Level III metastasis	1.202 (1.050–1.811)	0.014
Level IV metastasis	1.148 (1.031–1.717)	0.008

*LNCM* Lymph Node between superficial layer of deep Cervical fascia and deep fascia of infrahyoid Muscles

### Complications

The median follow-up time was 21.7 months (range 15–41). 67 (3.2%) of 2104 patients had voice changes, all of whom recovered within 1–6 months. Temporary vocal cord paralysis was confirmed in 46 patients (2.2%) by laryngoscope, and thirteen permanent hypocalcemia (0.4%) was observed after surgery.

## Discussion

In order to achieve the best chance of cure and effective disease control, thoroughness of dissection has to be taken into account. We prospectively performed comprehensive CND for PTC patients who underwent thyroidectomy and CND. In addition, data were analyzed for 2104 PTC patients to investigate the clinicopathologic characteristics for LNCM metastasis. The occurrence rate of LNCM was 21.4% (451/2104), and 68 (15.1%) of the 451 patients harbored metastatic LNCM. In total, the positive rate of the LNCM was 3.2% (68/2104). In this study, multivariate analysis revealed that a primary site in the inferior pole, ETE, level III and level IV metastasis were of higher LNCM metastasis rate, which was consistent with the findings by the previous report of lymph node metastasis between sternocleidomastoid and sternohyoid muscle [9].

Several studies have emphasized the importance of similar compartment in neck dissection for thyroid carcinoma. Sun et al. pioneered the confirmation of the significant involvement of lymph node metastasis between sternocleidomastoid and sternohyoid muscle (LNSS) in lateral neck dissection [9], which anatomically classified as part of the space of Burns. They concluded that the positive rate of LNSS was 22.6% in clinically node-positive (cN+) PTC, which was correlated with a primary site in the inferior pole, the lateral nodal metastasis, level III and level IV nodal metastasis [9]. Then, Homma et al. [10] reported two cases of PTC patients with level III and IV lymph node metastases as well as metastasis in the suprasternal space. Yu et al. [11] investigated the clinical significance of the suprasternal space lymph node (SSLN) in pathological node-positive (pN+) PTC patients. They concluded that metastasis rate of SSLN was 20.7% and the high SSLN metastasis of PTC was correlated with primary cancer site in the inferior thyroid pole, strap muscle invasion, level IV metastasis and LNSS metastasis. In our experience, LNCM was rarely occurred in the central neck compartment (21.4%), and the positive LNCM in PTC patients was infrequent as well (3.2%). Notably, among the 2348 patients with pN+ PTC, the metastasis rate of LNCM was 4.1%, which was much lower than the metastasis incidence of SSLN (20.7%) described by Yu et al. [11] According to their results, one fifth patients with pN+ PTC were performed incomplete CND and remained metastatic lymph nodes.

**Fig. 2:**
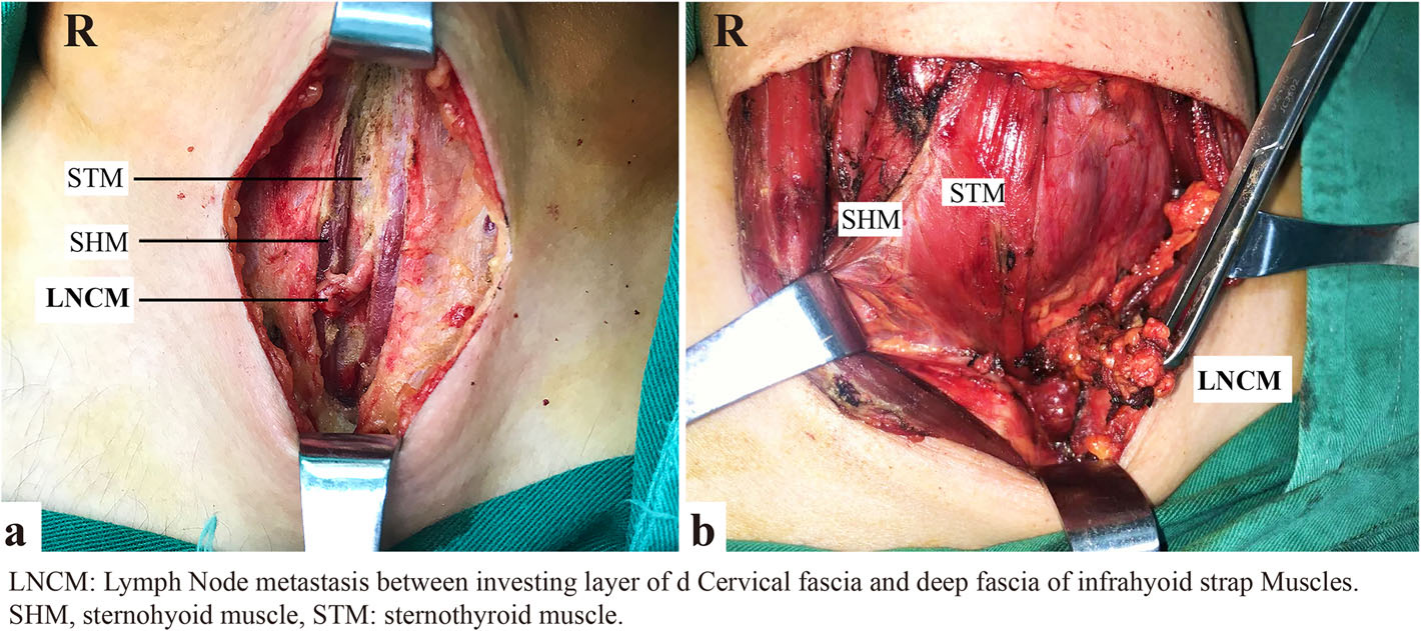
LNCM coverage area in vivo. Lymph nodes between sternohyoid and sternothyroid muscles (**a**) and lymph nodes in the suprasternal space (**b**)

The total number of lymph nodes in the central neck can range from 3 to 42 [19]. There is no consensus on the number of nodes removed or examined that would constitute an adequate dissection. Aimed to allow surgeons to more accurately report the extent of lymphadenectomy, we divide the central neck compartment into four subdivisions by deep fascia of infrahyoid strap muscles, pretracheal (visceral) fascial, and right RLN (Fig. 1). The proposed LNCM compartment is bounded superiorly by the hyoid bone, laterally by the carotid arteries, anteriorly by the investing layer of the cervical fascia, and posteriorly by the deep fascia of infrahyoid strap muscles, which is defined as Level VI 1st. In the current study, suprasternal space composed part of the LNCM (Fig. 1). Compared to SSLN reported by Yu et al. [11], LNCM encompasses lymph nodes in the suprasternal space and lymph nodes between sternohyoid and sternothyroid muscles (Figs. 1 and 2). LNCM can fall under the normal subdivisions of the central compartment. Subdivisions can actually record the extent of the CND, which is able to provide detailed information for the possible second operation.

Including LNCM as an anatomical part of the central neck allows for removal of previously unrecognized micrometastatic disease in 3.2% of PTC patients with the inferior portion lesions, ETE, level III and level IV metastasis. Dissection of the LNCM space is less invasive and easy to achieve and is not time-consuming. It is at the entrance of central neck compartment, which is easy to expose and has low risk of damaging RLN or parathyroid. With the application of intraoperative neuromonitoring and in situ preservation or auto-transplantation of parathyroid, the occurrence of vocal cord paralysis (2.2%) and permanent hypoparathyroidism (0.4%) in the current study were lower in this study [18, 20]. Therefore, in cases where LNCM space metastasis is suspected or preoperative ultrasound and CT suggests LNCM metastasis, greater attention should be paid to the nodal tissue in the LNCM space in thyroid carcinoma patients. These patients might benefit from a reduced risk of regional recurrence, central neck reoperative morbidity, and improved decision making in relation to the use of radioiodine ablation.

There are several limitations in the present study. The retrospective design is a limitation of the study. And this was two tertiary referral centers retrospective review and routine prophylactic nodal surgery was offered in China, however it is not standard elsewhere in the world, which is a major limitation. A prospective randomized trial with a long time follow-up period may help to further evaluate the clinical significance of LNCM in PTC patients.

## Conclusions

In summary, additional dissection of nodes in the LNCM were accessible and might not increase morbidity. Therefore, attention should be paid to the lymph tissue between investing layer of cervical fascia and deep fascia of infrahyoid strap muscles for PTC patients, especially in presence of inferior portion lesions, ETE, level III and level IV metastasis.

## Data Availability

Not applicable.
